# Influence of amount and percentage of CXCR4-using virus in predicting week 48 responses to maraviroc in treatment-naïve patients

**DOI:** 10.1186/1758-2652-13-S4-O11

**Published:** 2010-11-08

**Authors:** H Valdez, D Chapman, P Biswai, M Lewis, C Craig, J Heera, S Ellery, LC Swenson, PR Harrigan

**Affiliations:** 1 Pfizer Inc, New York, USA; 2 Pfizer Inc, Collegeville, USA; 3 Pfizer Global Research and Development, Sandwich, UK; 4 Pfizer Global Research and Development, New London, USA; 5 BC Centre for Excellence in HIV/AIDS, Vancouver, Canada

## Background

Both population and ultra-deep sequencing (UDS) of the HIV-1 V3 loop are useful in selecting candidates for maraviroc (MVC) therapy. We used mathematical modeling to determine that patients whose non-R5 HIV comprises <2% of the viral population by UDS are likely to respond to a MVC-containing regimen. However, the predictive value of absolute amount of non-R5 HIV is unknown.

## Objective

To determine whether non-R5 viral load contributes to predicting response to a MVC-containing regimen.

## Methods

Patients enrolled in the MERIT study (MVC or efavirenz plus zidovudine/lamivudine in treatment-naïve patients) with R5 virus at screening (by original Trofile assay) and randomized to the twice-daily MVC arm were included. UDS was performed with a 454/Roche GS-FLX instrument. Tropism was predicted using the "geno2pheno" co-receptor algorithm (g2p). A sample was considered R5 if <2% of variants had a score below 3.5 FPR. MVC responses at Week 48 were predicted by descriptive statistics and mathematical modeling.

## Results

Samples for 343 patients (308 R5, 35 non-R5) were available. Baseline median CD4 and mean viral load (VL) were 247 and 232 cells/µL and 4.9 and 4.6 log10 c/mL in patients with R5 and non-R5 virus. No CXCR4-using viruses were detected in 249/343 (73%) patients. Among the 94 patients with detectable CXCR4-use, median (q25, q75) percent and absolute levels of CXCR4-using viruses were 0.8% (0.4-8.1) and 2.9 (2.3-3.5) log10 c/mL, respectively. Week 48 virologic responses are shown in Table 1.

**Table 1:** 

Baseline level of CXCR4-using virus	<50 HIV-1 RNA c/mL at Week 48, n/N (%)
Percentage	

<2%	207/308 (67.2)

2%—<10%	7/13 (53.8)

≤10%	11/22 (50.0)

Amount (log10 copies/mL)	

<1.0	171/251 (68.1)

1.0—<2.0	12/15 (80.0)

2.0—<3.0	23/36 (63.9)

3.0—<4.0	13/25 (52.0)

≤4.0	6/16 (37.5)

In univariate models, baseline CD4 and percent of CXCR4-using virus were not significant predictors of week 48 response (p=0.12; p=0.26); VL and absolute amount of CXCR4-using virus were significant (p=0.02; p=0.03) and were included in the multivariate model (p=0.02 for both in final model).

## Conclusion

In MVC-treated patients in the MERIT study, baseline VL and absolute amount of CXCR4-using virus were predictive of Week 48 response. It is possible that total burden of CXCR4-using virus in drug-naive individuals may play a greater role than the percentage of such virus in predicting response to regimens containing a CCR5 antagonist.

